# 811. Impact of the COVID-19 Pandemic on Surgical Volume and Surgical Site Infections (SSI) in a Large Network of Community Hospitals

**DOI:** 10.1093/ofid/ofab466.1007

**Published:** 2021-12-04

**Authors:** Erin Gettler, Jessica Seidelman, Becky A Smith, Deverick J Anderson

**Affiliations:** 1 Duke University School of Medicine, Durham, NC; 2 Duke University, Durham, NC; 3 Duke Center for Antimicrobial Stewardship and Infection Prevention, Durham, NC

## Abstract

**Background:**

The COVID-19 pandemic significantly impacted hospitalizations and healthcare utilization. Diversion of infection prevention resources toward COVID-19 mitigation limited routine infection prevention activities such as rounding, observations, and education in all areas, including the peri-operative space. There were also changes in surgical care delivery. The impact of the COVID-19 pandemic on SSI rates has not been well described, especially in community hospitals.

**Methods:**

We performed a retrospective cohort study analyzing prospectively collected data on SSIs from 45 community hospitals in the southeastern United States from 1/2018 to 12/2020. We included the 14 most commonly performed operative procedure categories, as defined by the National Healthcare Safety Network. Coronary bypass grafting was included a priori due to its clinical significance. Only facilities enrolled in the network for the full three-year period were included. We defined the pre-pandemic time period from 1/1/18 to 2/29/20 and the pandemic period from 3/1/20 to 12/31/20. We compared monthly and quarterly median procedure totals and SSI prevalence rates (PR) between the pre-pandemic and pandemic periods using Poisson regression.

**Results:**

Pre-pandemic median monthly procedure volume was 384 (IQR 192-999) and the pre-pandemic SSI PR per 100 cases was 0.98 (IQR 0.90-1.04). There was a transient decline in surgical cases beginning in March 2020, reaching a nadir of 185 cases in April, followed by a return to pre-pandemic volume by June (figure 1). Overall and procedure-specific SSI PRs were not significantly different in the COVID-19 period relative to the pre-pandemic period (total PR per 100 cases 0.96 and 0.97, respectively, figure 2). However, when stratified by quarter and year, there was a trend toward increased SSI PR in the second quarter of 2020 with a PRR of 1.15 (95% CI 0.96-1.39, table 1).

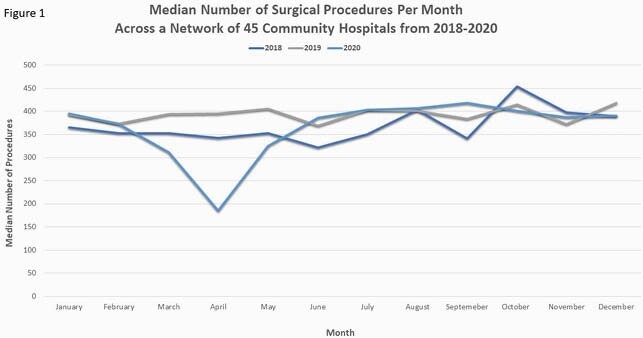

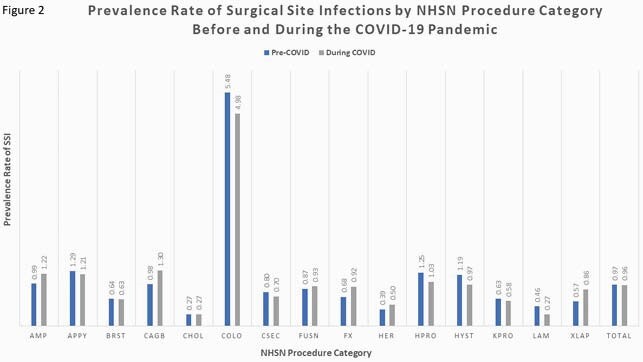

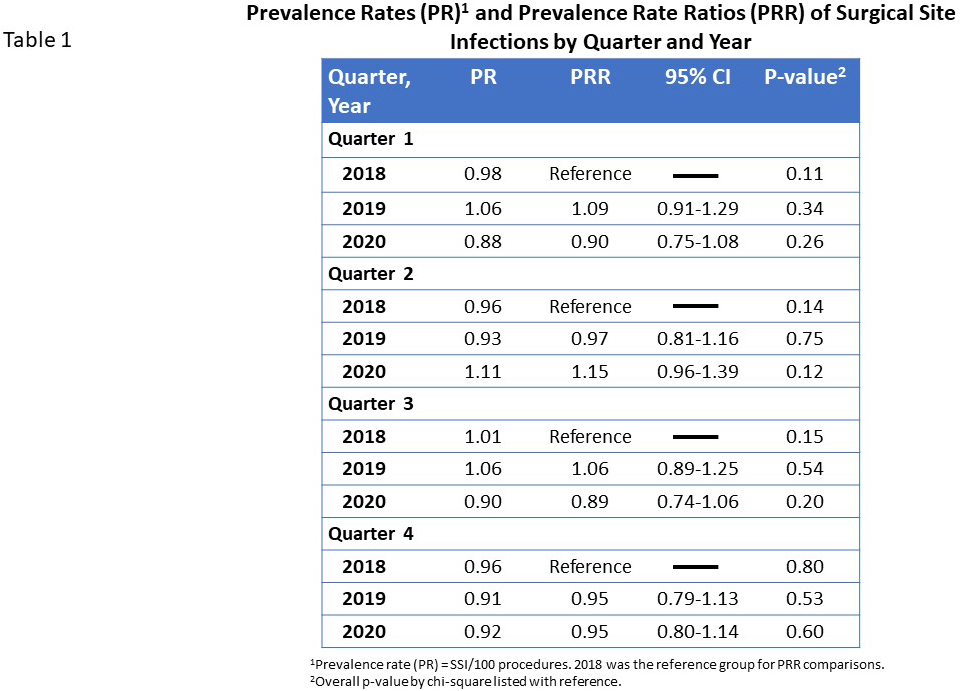

**Conclusion:**

The decline in surgical procedures early in the pandemic was short-lived in our community hospital network. Although there was no overall change in the SSI PR during the study period, there was a trend toward increased SSIs in the early phase of the pandemic (figure 3). This trend could be related to deferred elective cases or to a shift in infection prevention efforts to outbreak management.

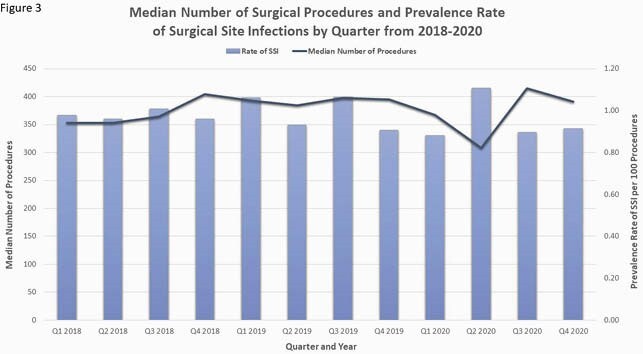

**Disclosures:**

**All Authors**: No reported disclosures

